# Effect of AMPK signal pathway on pathogenesis of abdominal aortic aneurysms

**DOI:** 10.18632/oncotarget.21608

**Published:** 2017-10-07

**Authors:** Le Yang, Lin Shen, Peixian Gao, Gang Li, Yuxiang He, Maohua Wang, Hua Zhou, Hai Yuan, Xing Jin, Xuejun Wu

**Affiliations:** ^1^ Department of Vascular Surgery, Shandong Provincial Hospital affiliated to Shandong University, Jinan 250012, China; ^2^ Department of Ophthalmology, Qilu Hospital of Shandong University, Jinan 250012, China

**Keywords:** AMPK, aneurysm, inflammation, neovascularization, MMP

## Abstract

**Background and aims:**

Determine the effect of AMPK activation and inhibition on the development of AAA (abdominal aortic aneurysm).

**Methods:**

AAA was induced in *ApoE*^−/−^ mice by Ang II (Angiotensin II)-infusion. AICAR (5-aminoimidazole-4-carboxamide-1-β-d-ribofuranoside) was used as AMPK activator and Compound C was used as AMPK inhibitor. We further investigate the effect of metformin, a widely used anti-diabetic drug which could activate AMPK signal pathway, on the pathogenesis of aneurysm.

**Results:**

Phospho-AMPK level was significantly decreased in AAA tissue compared with control aortas. AICAR significantly reduced the incidence, severity and mortality of aneurysm in the Ang II-infusion model. AICAR also alleviated macrophage infiltration and neovascularity in Ang II infusion model at day 28. The expression of pro-inflammatory factors, angiogenic factors and the activity of MMPs were also alleviated by AICAR during AAA induction. On the other hand, Compound C treatment did not exert obvious protective effect. AMPK activation may inhibit the activation of nuclear factor-κB (NF-κB) and signal transducer and activator of transcription-3 (STAT-3) during AAA induction. Administration of metformin also activated AMPK signal pathway and retarded AAA progression in Ang II infusion model.

**Conclusions:**

Activation of AMPK signaling pathway may inhibit the Ang II-induced AAA in mice. Metformin may be a promising approach to the treatment of AAA.

## INTRODUCTION

AAA (Abdominal Aortic Aneurysm) is the 10th leading cause of death among men over age 65. Open surgery and endovascular repair remained to be the only two established managements of AAA [1]. These two strategies were recommended when the maximal aortic diameter was greater than 5 cm [2]. If these managements were not applicable, AAA will grow larger and result in rupture, with high mortality. Although many studies have been carried out to investigate the pathogenesis of AAA, effective pharmacological therapies were still not available. As a result, physicians remain incapable to modify the progression of AAA [3].

AMPK (Adenosine monophosphate-activated protein kinase) is a member of a metabolite-sensing protein kinase family, which coordinates multiple metabolic pathways to balance energy demand and supply [4]. AMPK is a trimer consisting of one catalytic subunit (α) and two regulatory subunits (β and γ) [5]. In mammals, each subunit has multiple isoforms and expresses in different tissues. For example, there are two isoforms (α1 and α2) of catabolic α subunit, whereas two (β1 and β2) and three (γ1, γ2 and γ3) were present in β and γ subunit respectively [6]. Activation of AMPK attenuates anabolic processes such as the synthesis of proteins, fatty acids and cholesterol, and stimulates ATP (Adenosine Triphosphate) generating catabolic pathways such as respiration of glucose and fatty acids [7].

However, this metabolic regulatory effect of AMPK signal pathway is not the focus of this article, we dedicated to clarify the role of AMPK signal pathway in the pathogenesis of aneurysms. The activation of AMPK by metformin inhibits monocyte-macrophage differentiation and retards the pathogenesis of atherosclerosis [8]. This anti-inflammatory effect implies that AMPK activation may attenuate AAA progression considering AAA is mainly an inflammatory disease. Moreover, several recent studies reported the deletion of AMPKα1 or AMPKα2 exacerbated atherosclerosis in low density lipoprotein receptor knockout mice [9, 10]. However, according to another investigation, AMPKα2 activation by nicotine instigates formation of aneurysms in the Ang II (Angiotensin II) infusion model. In that study, AMPKα2^−/−^ApoE^−/−^ mice is resistant to aneurysmal formation [11]. These controversial results make it difficult to illuminate the relationship between pharmacological activation/inhibition of AMPK signal pathways and prognosis of AAA.

Ang II infusion model, a widely accepted mice model of aneurysm, [12] was adopted in this investigation. Two *in vivo* experiments were designed to evaluate the relationship between pharmacological activation/inhibition of AMPK and formation of AAA. AICAR (5-aminoimidazole-4-carboxamide-1-β-d-ribofuranoside) was used as AMPK activator and Compound C was used as AMPK inhibitor in the first *in vivo* study. Results of this study indicated AMPK activation may be beneficial to the prevention of AAA. Based on this, metformin, the most widely used anti-diabetic drug which had been reported to activate AMPK signal pathway, [13] was used in the second *in vivo* study to evaluate its pharmacological effects in AAA progression. Our results indicated AMPK activation could retard AAA progression and metformin may be used as a promising approach to the treatment of AAAs.

## RESULTS

### Decreased phospho-AMPKα levels and phospho-AMPKα/AMPKα ratios in aorta of AAA patients

**Figure 1:**
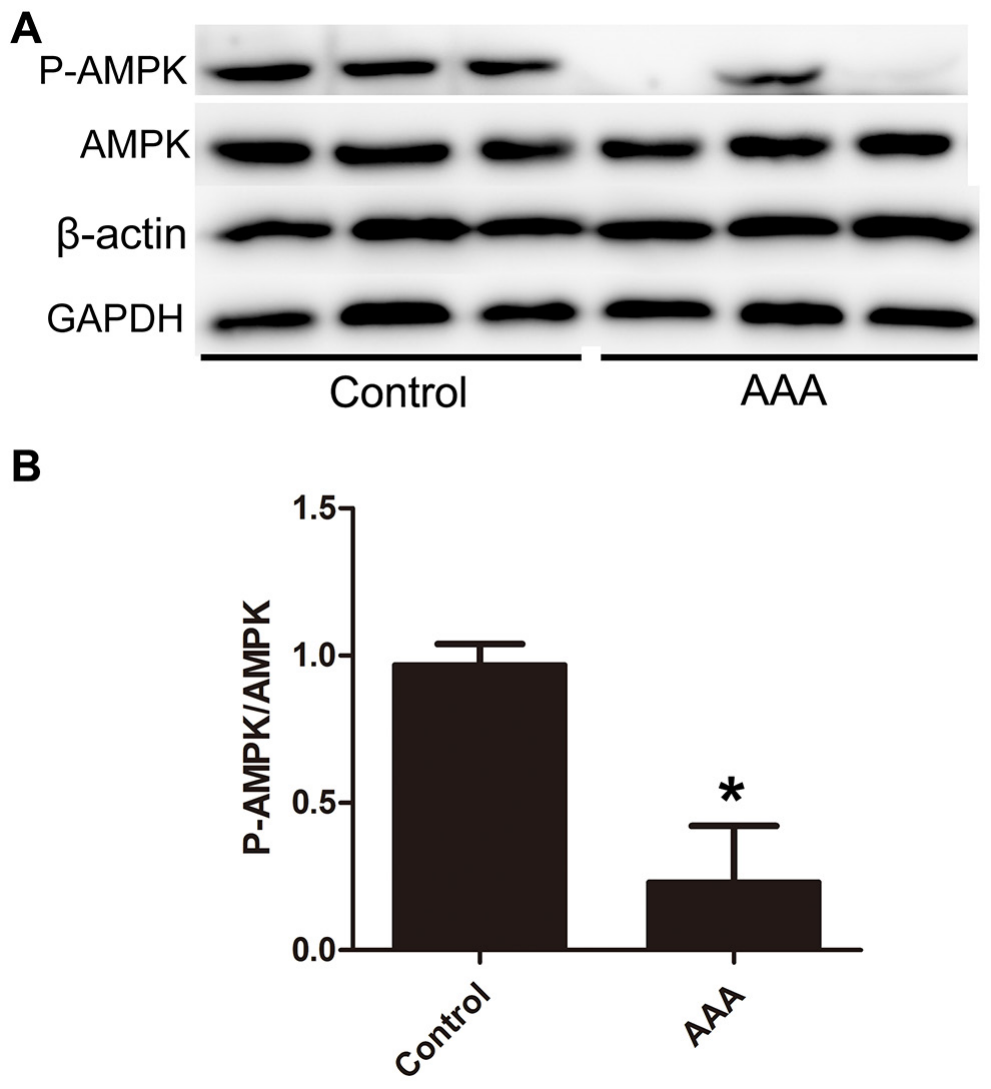
Decreased phospho-AMPK levels in the aortic wall of human abdominal aortic aneurysm (AAA) tissues **(A-B)**. Protein expression of P-AMPK, AMPK, β-actin and GAPDH of aneurysmal tissue and control aortic wall. Results are means± S.E.M. (*n* = 15 in AAA group, *n* = 8 in control group). ^*^P<0.05 vs. Control group.

The baseline clinical characteristics, diameter of aneurysm, and regular use of medication of the AAA patients were presented in the Supplementary Table 2. We firstly examined levels of phospho-AMPKα and total AMPKα in AAA tissues and control aortic tissues. Western blot analysis showed that there were no significant difference in total AMPKα protein levels between control aortic tissues and AAA tissues. On the other hand, phospho-AMPKα level was significantly lower in AAA tissues than in control aortic tissues, indicating the reduced activation of AMPKα signal pathway in human AAA. (Figure 1A-1B) The expression of phospho-AMPKα was readily detectable in the media of control aorta, whereas it was barely detectable in the AAA tissues. (Supplementary Figure 1)

### Effect of AMPK signal pathway on the incidence of AAA in Ang II induced AAAs

**Figure 2:**
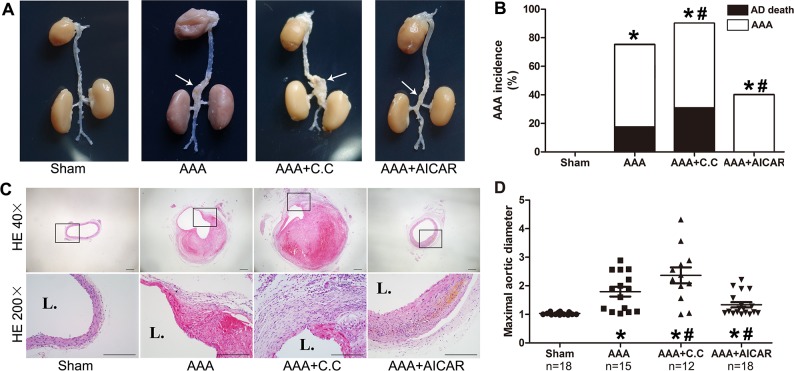
The activation of AMPK signal pathway retarded AAA progression while inhibition of AMPK signal pathway tended to aggravate AAA progression **(A)**. Representative macroscopic appearance of the aorta in 4 groups of mice who received treatment with PBS (sham group), Ang II (AAA group), Ang II plus Compound C (AAA+C.C group), Ang II plus AICAR (AAA+AICAR group), respectively. This figure is a representative figure of survival mice (n=18 in sham group, n=15 in AAA group, n=12 in AAA+C. C group, n=18 in AAA+AICAR group). The arrow indicated aneurysmal portion of each group. **(B)** Incidence and mortality of AAA in 4 groups of mice. **(C)** Representative hematoxylin and eosin staining in 4 groups of mice. **(D)** Maximal abdominal aortic diameters in all survival mice of 4 groups of mice. Scale bar indicated 200 μm. Results are means± S.E.M.^*^P<0.05 vs. Sham group; ^#^P<0.05 vs. AAA group.

Ang II infusion elevated the HR (heart rate) and BP (blood pressure) of mice modestly, while compound C and AICAR had no significant effect on the BM (body mass), HR, and BP (Supplementary Table 3). Maximal aortic diameters were increased in AAA, AAA+C. C (AAA+compound C) and AAA+AICAR groups. The aneurysmal incidence, severity and mortality were significantly lower in AAA+AICAR group compared with AAA group. On the other hand, the maximal aortic diameter in AAA+C. C group was slightly larger than AAA group. (Figure 2A-2B, 2D)

### Effect of AMPK signal pathway on Ang II induced morphological and histological changes in ApoE^−/−^ mouse aortas

**Figure 3:**
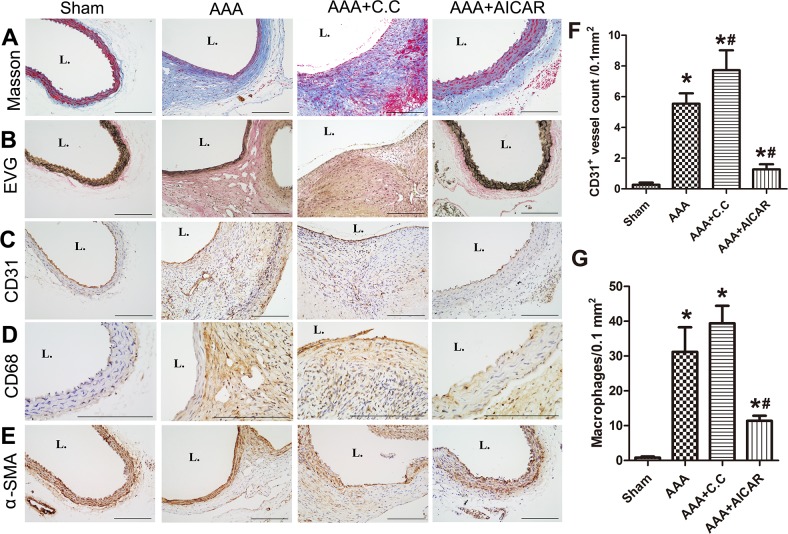
AMPK activation alleviated pathological features of AAA **(A-B)** Representative photomicrographs of Masson trichrome staining, elastic Van Gieson (EVG) staining of 4 groups of mice. (n=5 in each group used in pathological experiment) **(C-E)** Representative photomicrographs of CD31, CD68, α-SMA, staining of abdominal aortas of 4 groups of mice. Scale bar indicated 200 μm. **(F-G)** Quantitative analysis of CD31 positive vessels and CD68 positive cells in different groups. Results are means± S.E.M. ^*^P<0.05 vs. Sham group; ^#^P<0.05 vs. AAA group.

HE staining revealed destruction of the media and thickening of the adventitia in the AAA group compared with the sham group. These pathological features were aggravated in the AAA+C. C group and alleviated in the AAA+AICAR group. (Figure 2C) The relative contents of collagen and SMCs in the aortic wall were substantially increased in the AAA+AICAR group, whereas the content of macrophages and microvessels was decreased in the AAA+AICAR group. In contrast, these parameters showed only insignificant difference between the AAA group and AAA+C. C group. (Figure 3A-3E) Immunohistochemistry after polyclone antibodies pre-absorption with relative full-length antigens diminishes their labelling in mice aneurysmal tissue sections. (Supplementary Figure 2A-2C) The goat-anti-mouse negative control showed little positive staining area in all groups. (Supplementary Figure 3A)

### Effect of AMPK signal pathway on the expression of proinflammatory cytokines, vascular growth cytokines and the activity of metal matrix proteinase

**Figure 4:**
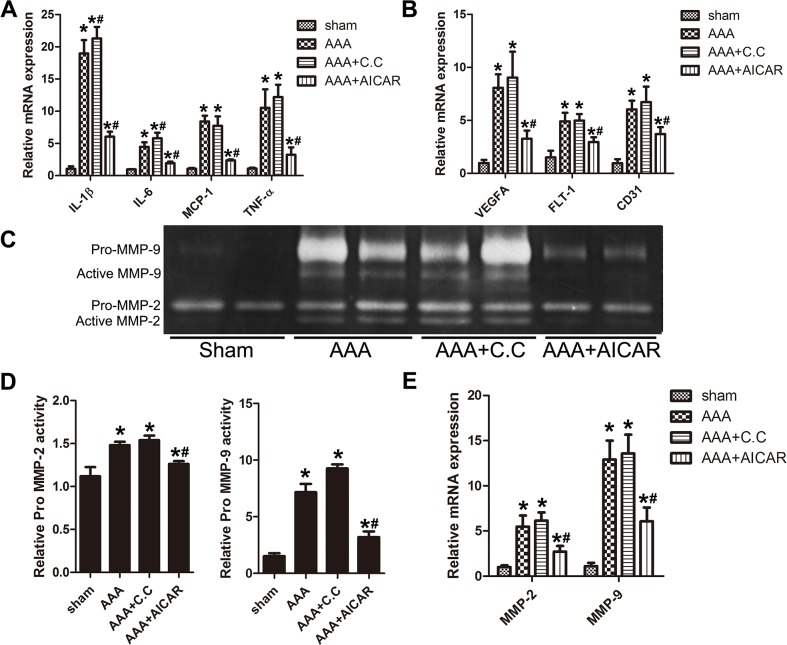
Quantitative RT-PCR analysis, gelatin zymography analysis of different groups of mice **(A)** Expression of IL-1β, IL-6, MCP-1 and TNF-α in abdominal aorta of 4 groups of mice. **(B)** Expression of VEGFA, Flt-1 and CD31 in abdominal aortas of 4 groups of mice. **(C)** Gelatin zymography of MMP-2 and MMP-9 activity and their quantitative analysis **(D)**. **(E)** Expression of MMP-2 and MMP-9 in abdominal aortas of 4 groups of mice. Results are means± S.E.M. (*n* =5 in each group) ^*^P<0.05 vs. Sham group; ^#^P<0.05 vs. AAA group.

As chronic inflammation of the aortic wall is a main feature of AAA, we examined the expression of proinflammatory cytokines, such as IL (interleukin)-1β, IL-6, MCP (monocyte chemotactic protein)-1 and TNF (tumor necrosis factor)-α. Expression of these cytokines in the AAA+AICAR group was significantly lower than those in the AAA group. While in the AAA+C. C group, expression of those proinflammatory cytokines was slightly higher. (Figure 4A) Neovascularization is also of vital importance in the AAA progression. VEGFA (vascular endothelial growth factor A), Flt (fms related tyrosine kinase)-1 and CD31(cluster of differentiation31) are the major vascular growth factors in the AAA progression. PCR analysis revealed the expression of these cytokines were elevated after AAA induction and this elevation could be attenuated by AICAR.(Figure 4B) IHC staining showed a reduced MMP-2 and MMP-9 positive area in AAA+AICAR group compared with AAA group (Supplementary Figure 3B-3C). The abundance of zymographic active form of MMP-2 and MMP-9 was increased in AAA group compared with Sham group. The activity of MMP-2 and MMP-9 were attenuated by AICAR. (Figure 4C-4D) PCR analysis also showed a reduced MMP-2 and MMP-9 mRNA level in AAA+AICAR group compared with AAA group. (Figure 4E) Compound C, on the other hand, aggravated the expression and activity of MMPs in AAA mice.

### STAT-3 and NF-κB signaling were involved in the effect of AMPK signal pathway on Ang II–induced AAA

**Figure 5:**
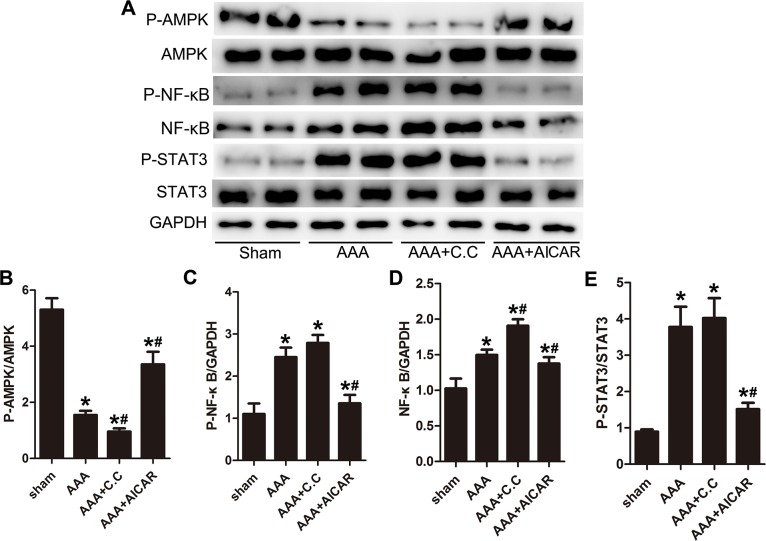
AMPK signal pathway correlated with NF-κB and STAT-3 signal pathways during AAA progression **(A)**, Representative Western blot analysis of phospho-AMPK (P-AMPK), AMPK, phospho-nuclear factor-κB (P-NF-κB), NF-κB, phospho-STAT3 and STAT3 in abdominal aortas of 4 groups of mice. **(B-C)** Quantitative analysis of P-AMPK normalized to AMPK protein level and P-NF-κB expression normalized to GAPDH level. **(D-E)** Quantitative analysis of NF-κB normalized to GAPDH level and P-STAT3 expression normalized to STAT3 level. Results are means± S.E.M. (*n*= 5 in each group) ^*^P<0.05 vs. Sham group;^#^P<0.05 vs. AAA group.

To verify the signaling proteins, we examined the expression level of AMPK, STAT-3 and NF-κB signal pathway. In AAA mice, Phospho-AMPK production was significantly decreased while NF-κB and Phospho-STAT-3 were increased compared with the sham group mice. AICAR group activate AMPK phosphorylation in AAA mice. AAA+AICAR group showed a lower level of Phospho-STAT-3 and NF-κB expression compared with AAA group. (Figure 5A-5E) AAA+C. C group downregulate AMPK phosphorylation while upregulate the NF-κB expression and STAT-3 phosphorylation. (Figure 5A-5E) In conclusion, there may be a causal reciprocal relationship between AMPK signal pathway and NF-κB and STAT3 signal pathway.

### Metformin downregulate on the incidence of AAA in Ang II infused mice

**Figure 6:**
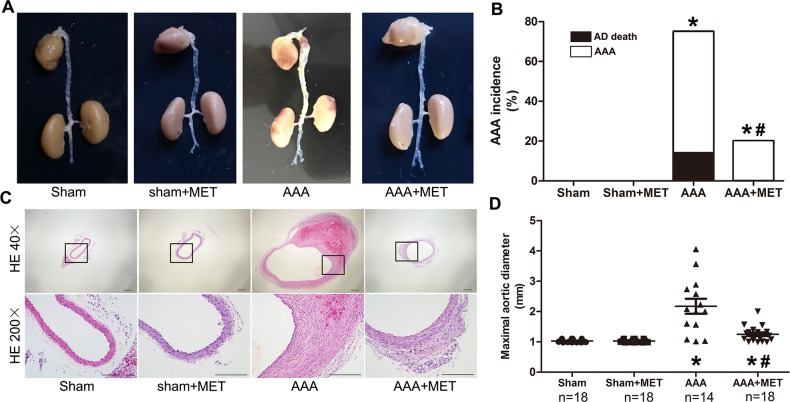
Orally administration of metformin retarded AAA progression in Ang II infusion model **(A)**. Representative macroscopic appearance of the aorta in 4 groups of mice who received treatment with PBS, Metformin (MET), Ang II, Ang II plus metformin, respectively. This figure is a representative figure of survival mice (n=18 in sham group, n=18 in Sham+MET group, n=14 in AAA group, n=18 in AAA+MET group). **(B)** Incidence and mortality of AAA in 4 groups of mice. **(C)** Representative hematoxylin and eosin staining in 4 groups of mice. **(D)** Maximal abdominal aortic diameters in 4 groups of mice. Scale bar indicated 200 μm. Datas are means± S.E.M. ^*^P<0.05 vs Sham group; ^#^P<0.05 vs AAA group.

Metformin, a widely used anti-diabetic drug which could activate AMPK signal pathway *in vivo*, was further evaluated in this study. Metformin (300mg/kg in daily drinking water) reduced the incidence, severity and mortality of AAA. (Figure 6A-6B, 6D) Absolute aortic growth was significantly less in metformin treated mice.

### Effect of metformin on ang II induced morphological and histological changes in ApoE^−/−^ mouse aortas

**Figure 7:**
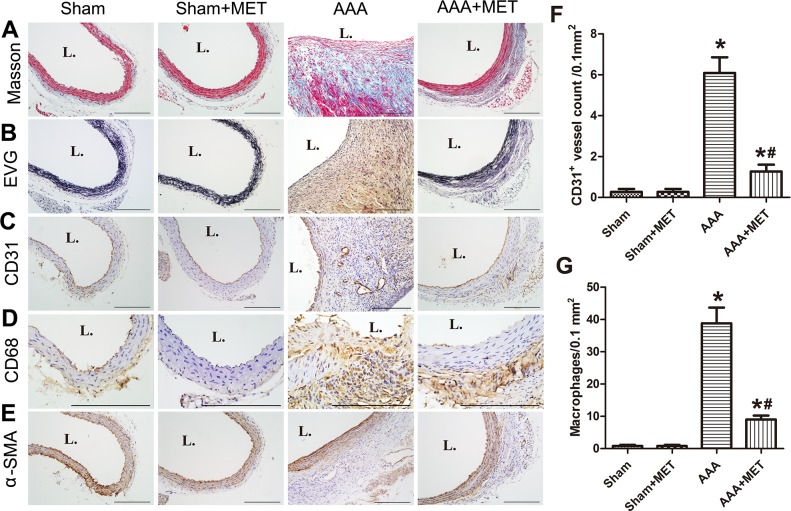
Orally administration of metformin alleviated pathological features of AAA **(A-B)** Representative photomicrographs of Masson trichrome, elastic Van Gieson (EVG) (n=5 in each group used for histological experiment), **(C-E)** Representative photomicrographs of CD31, CD68, α-SMA staining of abdominal aortas of 4 groups of mice. Scale bar indicated 200 μm. **(F-G)** Quantitative analysis of CD31 positive vessels and CD68 positive cells in 4 groups of mice. Results are means± S.E.M. ^*^P<0.05 vs. Sham group; ^#^P<0.05 vs. AAA group.

Metformin reduced the destruction of media and the marked thickening of adventitia. (Figure 6C) Metformin treatment also preserved the collagen and SMCs in the wall of aneurysm. On the other hand, macrophages and microvessels in the aneurysmal wall were reduced in the metformin treatment group. (Figure 7A-7E)

### Metformin downregulates the expression of proinflammatory cytokines, vascular growth cytokines and the activity of metal matrix proteinase

**Figure 8:**
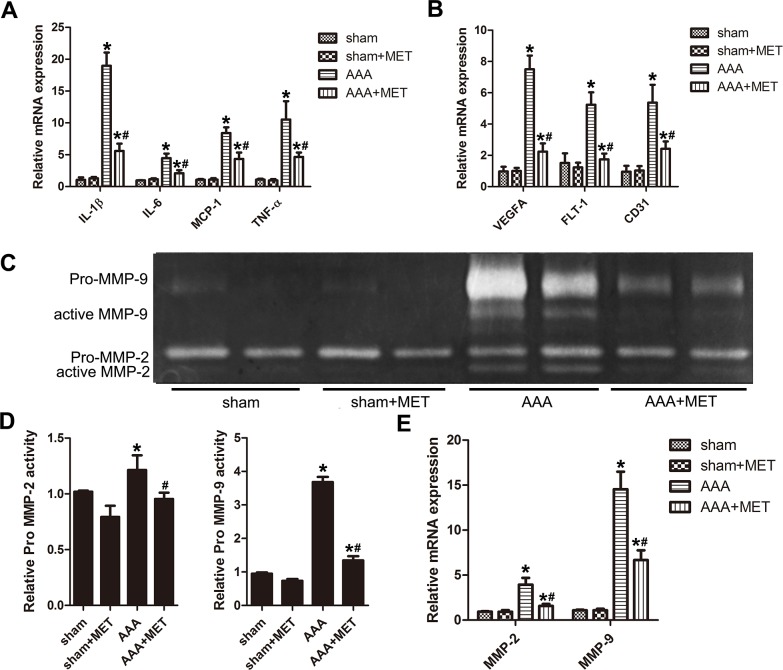
Quantitative RT-PCR analysis, gelatin zymography analysis of different groups of mice **(A)** Expression of IL-1β, IL-6, MCP-1 and TNF-α in abdominal aortas of 4 groups of mice. **(B)** Expression of VEGFA, Flt-1 and CD31 in abdominal aortas of 4 groups of mice. **(C)** Gelatin zymography of MMP-2 and MMP-9 activity and their quantitative analysis **(D)**. **(E)** Expression of MMP-2 and MMP-9 in abdominal aortas of 4 groups of mice. Results are means± S.E.M. (*n* = 5 in each group) ^*^P<0.05 vs. Sham group; ^#^P<0.05 vs. AAA group.

Compared with the AAA group, metformin inhibited the expression of proinflammatory cytokines such as IL-1β, IL-6, MCP-1 and TNF-α. (Figure 8A) Administration of metformin also inhibited the expression of vascular growth cytokines such as VEGFA, Flt-1 and CD31. (Figure 8B) Gelatin zymography analysis revealed reduced MMP-2 and MMP-9 activity after metformin treatment. (Figure 8C-8D) PCR analysis also showed a reduced MMP-2 and MMP-9 mRNA level in AAA+MET group compared with AAA group. (Figure 8E) IHC staining showed a reduced MMP-2 and MMP-9 positive area in AAA+MET group compared with AAA group (Supplementary Figure 4A-4B).

### STAT-3 signaling and NF-κB signaling were involved in the effect of metformin on Ang II–induced AAA

**Figure 9:**
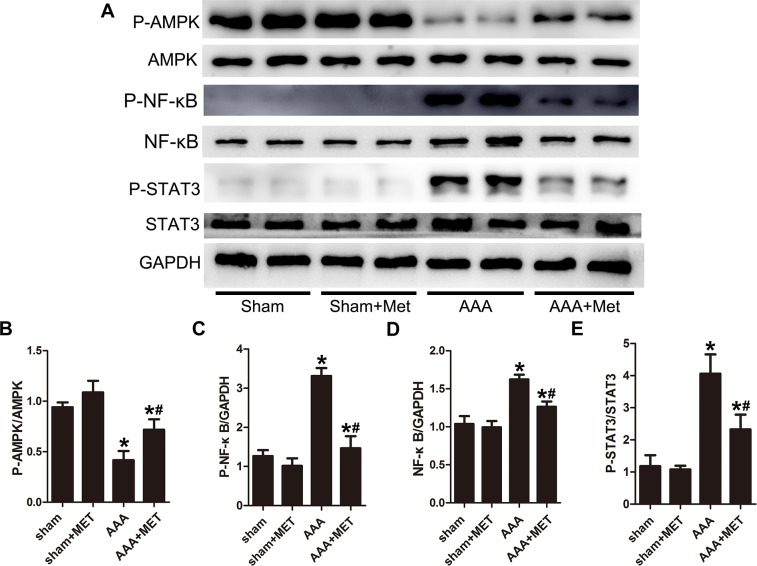
Metformin decreased the activity of NF-κB and STAT-3 signal pathway during AAA progression **(A)**, Representative Western blot analysis of Phospho-AMPK, AMPK, Phospho -NF-κB, NF-κB, Phospho -STAT3 and STAT3 in abdominal aortas of 4 groups of mice. **(B-C)** Quantitative analysis of Phospho -AMPK normalized to AMPK protein level and Phospho -NF-κB expression normalized to GAPDH level. **(D-E)** Quantitative analysis of NF-κB normalized to GAPDH level and P-STAT3 expression normalized to STAT3 level. Results are means± S.E.M. (*n* = 5 in each group) ^*^P<0.05 vs. Sham group;^#^P<0.05 vs. AAA group.

In AAA mice, Phospho-AMPK production was significantly decreased while NF-κB and phospho-STAT-3 were increased compared with the sham group mice. (Figure 9A-9E) Metformin treatment activated AMPK phosphorylation in AAA mice. After metformin treatment, the NF-κB expression and STAT-3 phosphorylation were downregulated. (Figure 9A-9E).

## DISCUSSION

To the best of our knowledge, this is the first study in the literature to report the pharmacological activation/inhibition of AMPK signal pathway and pathogenesis of abdominal aortic aneurysm. Human tissue study suggested that AMPK signal pathway seems to be inhibited in AAA patients. The *in vivo* study demonstrated that activation of AMPK signal pathway by AICAR reduced the incidence, severity and mortality of aneurysm, while the inhibition of AMPK signal pathway by Compound C tended to aggravate AAA progression. Activation of AMPK signal pathway seems to be associated with the inhibited NF-κB and STAT-3 signal pathway, which are of vital importance in the chronic inflammation in the AAA progression. Moreover, we illustrated that metformin, which had proven to be an AMPK activator, significantly attenuated Ang II-induced aortic aneurysm in ApoE^−/−^ mice. These results indicated that metformin could be a potential pharmacological therapy in AAA.

All three major cell types (endothelial cells, VSMC, and monocytes/macrophages) in the vasculature express AMPKα. AMPKα1 is the major isoform in vasculature cells is where as AMPKα2 is the minor isoform [10]. Previous studies illustrated AMPK signal pathway (α1 and α2) confers protection to various cardiovascular diseases, such as atherosclerosis, atherosclerotic vulnerability and atherosclerotic calcification [9, 10, 14]. Another study reported AMPKα2 could be activated by nicotine and initiate MMP-2 secretion in VSMCs. On the other hand, AMPKα2 knockout confers protection to AAA formation [11]. These controversial reports make it difficult to evaluate AMPK signal pathway in the development of aneurysm. We firstly observed significantly reduced levels of Phosphate-AMPKα in human AAA tissues than in control aortas. This clinical tissue investigation indicated that impairment of AMPK signal pathway may promote AAA formation. However, whether the activation of AMPK signal pathway exerts the protective effect on AAA formation remain elusive.

Although different isoforms of AMPKα could be evaluated by genetic editing method, pharmacological activation or inhibition of different AMPKα isoform remains impossible [15, 16]. Moreover, the strong homology between the AMPKα1 and AMPKα2 sequences in the vicinity of the substrate binding groove, suggests that the substrate specificities will be related [5]. Considering previously controversial results on the relationship between AMPK signal pathway and the formation of AAA, further studies of AMPK signal pathway in the aneurysm are needed to be carried out. In the first *in vivo* experiment, AICAR was used as AMPK activator and Compound C was used as AMPK inhibitor. AICAR is converted to 5-aminoimidazole-4-carboxamide ribonucleoside (ZMP) in the cell where it mimics AMP binding to AMPK increases its phosphorylation at threonine 172, leading to prolonged activation of AMPK signal pathway [17, 18]. Compound C, on the other hand, is widely used in the *in vitro* and *in vivo* assays as a selective AMPK inhibitor [19]. It is worth mention that AICAR or compound C might have effects on signaling pathways other than AMPK;[17, 20, 21] however, there are no known enzymatic pathways that are both stimulated by AICAR and inhibited by compound C, [6, 22] suggesting that the effects of AICAR or compound C were due to AMPK signal pathway.

Infiltration of macrophages, neovascularization and elastic degradation are the main pathological features of aneurysm [23, 24]. These three pathological changes are interdependent and of vital importance in the formation and progression of AAAs. Macrophages themselves secrete MMPs and VEGFs to promote neovascularization and elastic degradation [25, 26]. On the other hand, neovascularization and elastic degradation can facilitate macrophage's infiltration into the AAA portion [27, 28]. Several previous studies had reported that the activation of AMPK signal pathway could exert anti-inflammatory and anti-neovascularization effect in atherosclerosis, cancer and chronic kidney disease [9, 29, 30]. In this study, activation of AMPK signal pathway, markedly reduced macrophage accumulation in AAA tissues and significantly decreased the expression of proinflammatory cytokines, such as MCP-1, IL-1β, IL-6 and TNF-α. On the other hand, inhibition of AMPK signal pathway does not exert such beneficial effects. Moreover, AMPK activation also reduced the mural microvessels density and the expression of several angiogenic factors such as VEGFA, CD31 and Flt-1. These results indicate activation of AMPK may alleviate chronic inflammation and neovascularization in AAA tissue.

Several articles had discussed the possible molecular mechanisms underlying the relationship between the activity of AMPK signal pathway and the chronic inflammation in the macrophages, which is the main pathological feature in AAA. AMPK was first implicated in chronic inflammation in studies showing that AMPK activators diminish inducible iNOS (inducible nitric oxide synthases) synthesis in macrophages and adipocytes. [31] Subsequently, Sag et al. [32] reported that treatment of macrophages with anti-inflammatory cytokines, such as IL-10 and TGF-β, rapidly activated AMPK in these cells, whereas the proinflammatory stimulus LPS diminished AMPK activity. Likewise, inhibition of AMPK activity by RNAi (RNA interference) or transfection of an inactive AMPK mutant enhanced LPS-induced increases in the inflammatory cytokines TNF-α and IL-6 and diminished IL-10 in these cells. More recently, Yang and coworkers [33] demonstrated that increased AMPK activity, caused by the expression of a constitutively active AMPKα1, inhibits both LPS and palmitate-induced NF-κB signaling in macrophages. They also observed that inactivating AMPK in macrophages in a macrophage/adipocyte co-culture system inhibited both insulin signaling and glucose uptake in the adipocytes (i.e., it produced ischemia reperfusion injury). Finally, the same investigators found that AMPK activation increased SIRT1 (Sirtuin 1) expression in macrophages and that this led to the deacetylation and downregulation of NF-κB (i.e., decreased inflammation).

MMP-2 and MMP-9 work in concert to produce aortic aneurysms [34]. Previously study revealed that the activation of AMPKα2 by nicotine could initiate MMP2 secretion in VSMC to induced AAA in ApoE^−/−^ mice [11]. However, another study reported AMPKα2 deletion could induce plaque instability in ApoE^−/−^ mice [10]. In our study, MMP-2 activity does not alter significantly after AMPKα activation. There was a significantly reduced MMP-9 activity after the use of AMPK activators. The reduced MMP-9 activity may be associated with reduced aortic mural macrophage infiltration considering macrophages are the major sources of MMP-9 [34]. Besides this, previous studies reported AMPK activator may exert a direct prohibitive effect on MMP-9 secretion. The activation of AMPKα by metformin or other anti-oxidant agents can inhibit MMP-9 secretion in macrophages [8].

NF-κB and STAT-3 are the key proinflammatory transcription factors responsible for the regulation of the cytokine network in vasculature cells [35, 36]. Angiotensin II and other aneurysmal prone factors (such as nicotine and etc.) may activate NF-κB in smooth muscle cells and macrophages, which in turn promotes AAA development. The activities of NF-κB and STAT-3 also correlated with AAA severity [37]. STAT3 activation is necessary for Ang II-stimulated MMP secretion and the increase in total macrophages and the ratio of M1/M2 macrophages in suprarenal aortas of ApoE^−/−^ mice [8, 35]. In the present study, NF-κB and STAT-3 activation was significantly attenuated in AICAR group, suggesting that the effect of AMPK activation in AAA model was correlated with downregulating NF-κB and STAT-3 activation. However, because of the limited mice aortic tissue in each group, images of P-NF-κB and P-STAT3 for Sham and AAA in Figure 5A are quite inconsistent in those in Figure 9A.

Another important finding in this study is orally administration of metformin could delay the formation of aneurysm. Metformin, as the most commonly used anti-diabetic agents, has beneficial effects in cardiovascular complications besides glycemic control [8]. Earlier studies had shown that the pleiotropic benefits of metformin are in part mediated by the activation of AMPK [38]. Metformin had been proven to improve the lifespan and healthy span in mice via AMPK signal pathway [39]. Previous studies showed metformin could effectively inhibit PMA-induced monocyte-to-macrophage differentiation through the activation of AMPKα [8]. Another article reported metformin to suppress the enlargement of aneurysm in AAA patients [40]. Our experiment revealed metformin could suppress Ang-induced aneurysm progression, possibly by the activation of AMPK signal pathway. These results indicated that metformin may serve as an effective and safe drug to control AAA progression.

There were several limitations in this investigation. Firstly, all drugs including ACIAR [41, 42], Compound C [20, 42] and metformin [43] have off-target effects. Through this investigation, we can only delineate a potential link between the AMPK activation and the retard of aneurysmal growth. Genetic investigation may be beneficial to set aside the off-target effect of these chemical compounds. However, by using genetic approach, scientists can only evaluate role of AMPKα1 or AMPKα2 in the pathogenesis of aneurysm separately. Considering this, using chemical compound may be indispensible to evaluate the overall effect of AMPK signal pathway on the pathogenesis of aneurysms. Secondly, the *in vitro* study (cell experiment) was not included in this study. Many different types of cells, such as smooth muscle cells, [44] macrophages, [8] mast cells [45] and even perivascular adipose tissue [46] were involved in the development of aneurysm. The *in vitro* study using a specific cell type may be not sufficient to explain the overall mechanism of one drug in the development of aneurysm.

## MATERIALS AND METHODS

### Patients enrollment and tissue collection

Patients with aortitis, connective tissue disorders, or ruptured aneurysm were excluded. During surgery, aortic tissues were collected from the largest portion of the aneurysm, which were routinely excised and discarded during repair. Control abdominal aortic tissue was collected from 8 age-matched organ donors without aortic aneurysm, dissection, coarctation or previous aortic repair. Periaortic fat and intraluminal thrombus were trimmed away, and samples were rinsed with PBS. Samples were snap frozen and stored at –80°C for protein analysis. This study was approved by the Medical Ethics Committee of Shandong Provincial Hospital affiliated to Shandong University and was conducted in accordance with the Declaration of Helsinki. Informed consent was obtained from all participants.

### AAA model

The AAA model was induced in ApoE^−/−^ (apolipoprotein E deficient) mice (C57Bl6/J background) at the age of 8 to 10 weeks as described previously. Briefly, mice were implanted with Alzet osmotic minipumps (Model 1004, Durect Corporation), filled with Ang II solutions (Abcam, Cambridge, United Kingdom. Ab120183, 1,000 ng/kg/min) under anesthesia by intraperitoneal injection of chloralhydrate (30 mg/kg of body mass). The infusion persisted for up to 4 weeks. At indicated time point, mice were sacrificed for image acquisition and tissue harvest. All procedures were approved by the Animal Care and Use Committee of Shandong Provincial Hospital and were conducted following the institutional guidelines.

### Treatment with AMPK modulators in AAA model

Two *in vivo* experiments were designed to evaluate the relationship between pharmacological activation/inhibition of AMPK and the formation of AAA.

Experiment 1: Chemicals compounds and AAA progression.

To evaluate the relationship between AMPK activation and AAA formation, both AMPK activator and inhibitor were used. AICAR (5-aminoimidazole-4-carboxamide-1-β-d-ribofuranoside) was used as AMPK activator and Compound C was used as AMPK inhibitor. Mice were divided into four groups (n=18 in each group), one group was used as normal control (Sham group). Rest AAA mice were randomly given AICAR (500 mg/kg, i.p. q.d. AAA+ACIAR group), Compound C (300 mg/kg, i.p. q.d. AAA+C. C group) or equal volume of PBS (Phosphate Buffer solution, AAA group). These injections were started once the implantation of pumps and persisted 28 days.

Experiment 2: Metformin and AAA progression

In the second *in vivo* study, mice were randomly divided into four groups (n=18 in each group). One group was used as normal control (Sham group) and another group was given metformin (Sham+MET group) at a dose of 100 mg/kg/day in normal drinking water. Rest two groups were inducted to aneurysm. Among them, one group was given normal drinking water (AAA group) and another group was given metformin (AAA+MET group).

### Histological analysis

Mouse suprarenal aortas were fixed in 4% paraformaldehyde for at least 24 hours, paraffin-embedded, cut into 5-μm slices, and deparaffinized. HE staining, Elastic Van-Gieson (EVG) staining and masson-trichrome staining were performed by a standard protocol. Immunohistochemical (IHC) studies were performed following standard protocol using the anti-CD68 (cluster of differentiation 68) antibody (Ab955, dilution rate: 1:150), anti-CD31 (platelet/endothelial cell adhesion molecule 1) antibody (Ab38364, dilution rate: 1:50), anti-alpha-smooth muscle actin (α-SMA) antibody (Ab5694, dilution rate: 1:200), anti-MMP-2 antibody (Ab 37150, dilution rate: 1:200) and anti-MMP-9 antibody (Ab 38898, dilution rate: 1:500) [47]. Pre-absorption tests were performed to polyclone antibodies (Ab5694, Ab 37150, Ab 38898). Smooth muscle actin (Thermo Fisher, A12375), Matrix Metalloproteinases-2 recombined protein (Thermo Fisher, RP-77542) and Matrix Metalloproteinases-9 recombined protein (Thermo Fisher, RP-75655) were used in the pre-absorption tests. After primary incubation, slices were washed and incubated with the appropriate secondary antibody and stained with 3, 3-diaminobenzidine.

Medial smooth muscle density was calculated by dividing the histological-positive area by the total area. Number of CD 68-positive macrophages CD31-positive microvessels were measured by counting the total number in 5 grid fields composed of a 400×250-μm rectangle (0.1 mm^2^) in each mouse.

### qRT-PCR (quantitative real-time reverse-transcription polymerase chain reaction)

Total RNA was extracted from suprarenal aorta using Trizol Reagent (Invitrogen) according to the manufacturer's protocol. 1 μg sample of total RNA of the aorta was reverse-transcribed to cDNA (complimentary DNA) with the Strand cDNA Synthesis Kit (Takara). qRT-PCR analysis was performed using the Power SYBR Green PCR Mastermix (Takara) according to the manufacturer's protocol. Primers used in the PCR were described in the Supplementary Table 1.

### Western blot

Proteins were isolated from frozen suprarenal aortas using RIPA buffer (Beyotime Institute of Biotechnology, Shanghai, China) containing protease inhibitors. Protein extracts were separated by 10% sodium dodecyl sulfate-polyacrylamide gel electrophoresis and electrically transferred to polyvinylidene difluoride membranes, which were blocked with 10% nonfat dry milk in TBS-0.05% Tween 20 for 1 hour. Then, membranes were incubated overnight at 4°C using below primary antibodies: anti-Phospho-AMPKα (Thr172) antibody (2535, cell signaling technology, 1:1000), anti-AMPKα antibody (2603, cell signaling technology, 1:1000), anti-Phospho-NF-κB P65 (nuclear factor κB P65) (Ser536) antibody (3033, cell signaling technology, 1:1000), anti- NF-κB P65 antibody (4764, cell signaling technology, 1:1000), anti-Phospho -STAT3 (signal transducer and activator of transcription 3) (Tyr705) antibody (Ab76315, 1:20000), anti-STAT3 antibody (Ab119352, 1:1000), anti-β-actin antibody (sc-81178, Santa Cruz Biotechnology, Santa Cruz, Calif, 1:500) and anti-GAPDH antibody (ab-8245, 1:5000). After that, the appropriate secondary antibody was applied. Blots were detected using an ECL Prime Western Blotting Detection reagent (Millipore). The signals were quantified by ImageJ software.

### Gelatin zymography

Proteins were extracted from suprarenal aortas using RIPA buffer. Equal amounts of 10 μg proteins were electrophoresed on 10% sodium dodecyl sulfate-polyacrylamide gel containing 0.1% gelatin. After that, the gels were renatured in renaturing buffer: 50 mM Tris-HCl containing 100 mM NaCl and 2.5% Triton X-100. Then, they were incubated with developing buffer, 50 mM Tris-HCl containing 10 mM CaCl_2_ overnight. After that, they were stained with staining buffer (0.8% Brilliant Blue R) (Beyotime, Beijing, China), and distained in water. Band intensities were quantified by the use of ImageJ software.

### Statistical analyses

Continuous data were presented as means ±S.E.M. In comparisons of two treatment conditions, two-tailed Student *t* test was used for normally distributed data and Mann–Whitney nonparametric test for skewed data that deviate from normality. In comparisons of three or more treatment conditions, one-way analysis of variance with Bonferroni post hoc test was used for normally distributed data and Kruskal–Wallis nonparametric test for skewed data. The semi-quantification for elastin and SMC content was compared using a nonparametric Kruskal-Wallis test, followed by post hoc analysis (Dunn test). *P*<0.05 was considered to be statistically significant. All data analyses were calculated by GraphPad Prism version 6 (GraphPad Software, Inc.).

## CONCLUSION

Activation of AMPK signaling pathway inhibit in the Ang II-induced AAA formation in mice. Metformin may be a promising approach to the treatment of AAA.

## SUPPLEMENTARY MATERIALS FIGURES AND TABLES



## Abbreviations

AMPK: AMP activated protein kinase
AAA: abdominal aortic aneurysm
AICAR: 5-aminoimidazole-4-carboxamide1-β-D-ribofuranoside
MMP: matrix metalloproteinase
Ang: angiotensin
NF-κB: nuclear factor-κB
STAT-3: signal transducer and activator of transcription-3
IL: interleukin
MCP: macrophage chemotaxis protein
TNF: tumor necrosis factor
VEGF: vascular growth factors
CD: cluster of differentiation
Flt: fms related tyrosine kinase
EVG: elastic van gieson
HE: hematoxylin-eosin
SMA: smooth muscle actin
MET: metformin
HR: heart rate
BP: blood pressure
BM: body mass
IHC: immunohistological chemistry
WB: western blot
qRT-PCR: quantitative real-time reverse-transcription polymerase chain reaction
