# Supplementary material for: Effect of AMPK signal pathway on pathogenesis of abdominal aortic aneurysms
Source: Oncotarget. 2017 Oct 7;8(54):92827–40. doi: 10.18632/oncotarget.21608 (PMC5696225; doi:10.18632/oncotarget.21608)
Supplement: Supplementary file 1 [file oncotarget-08-92827-s001.pdf]

Effect of AMPK signal pathway on pathogenesis of abdominal aortic aneurysms

SUPPLEMENTARY MATERIALS

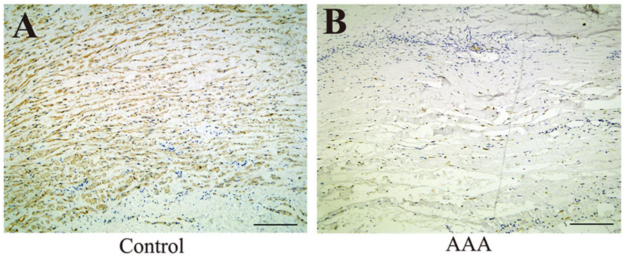

Supplementary Figure 1: Representative photomicrographs of P-AMPK staining of control aortic tissue **(A)** and AAA tissue **(B).** Scale bar indicated 200 µm.

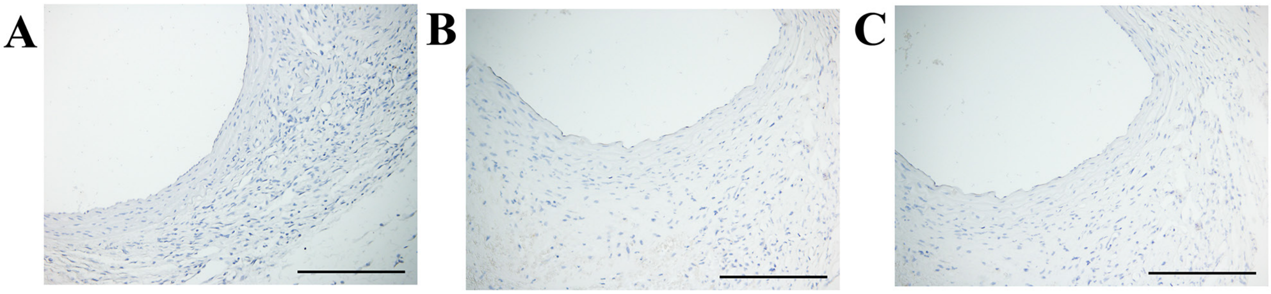

Supplementary Figure 2: Immunohistochemistry after polyclone antibodies pre-absorption with relative full-length antigens diminishes their labeling in mice aneurysmal tissue sections. (A) Ab5694 pre-absorbed with smooth muscle actin (Thermo Fisher, A12375); **(B)** Ab37150 pre-absorbed with Matrix Metalloproteinase-2 recombined protein (Thermo Fisher, RP-77542); **(C)** Ab38898 pre-absorbed with Matrix Metalloproteinase-9 recombined protein (Thermo Fisher, RP-75655).

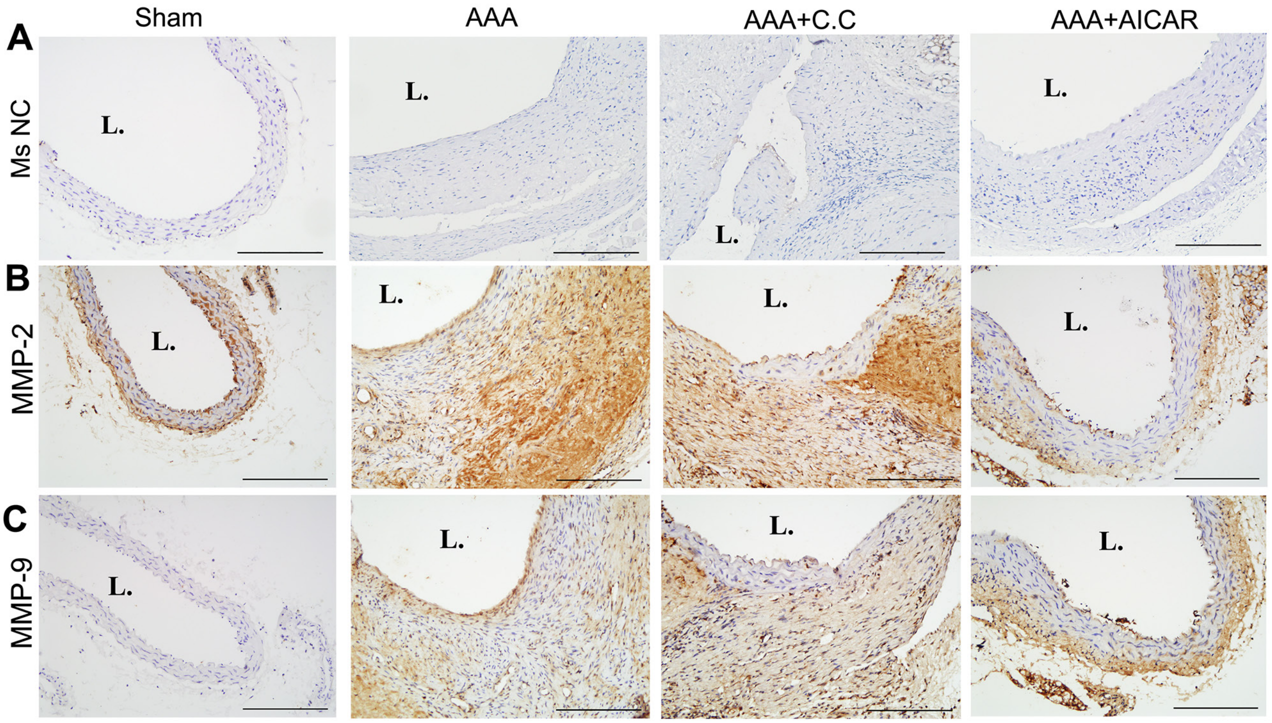

Supplementary Figure 3: Representative photomicrographs of mouse negative control **(A),** MMP-2 **(B),** MMP-9 **(C)** staining of abdominal aortas of 4 groups of mice. Scale bar indicated 200 μm.

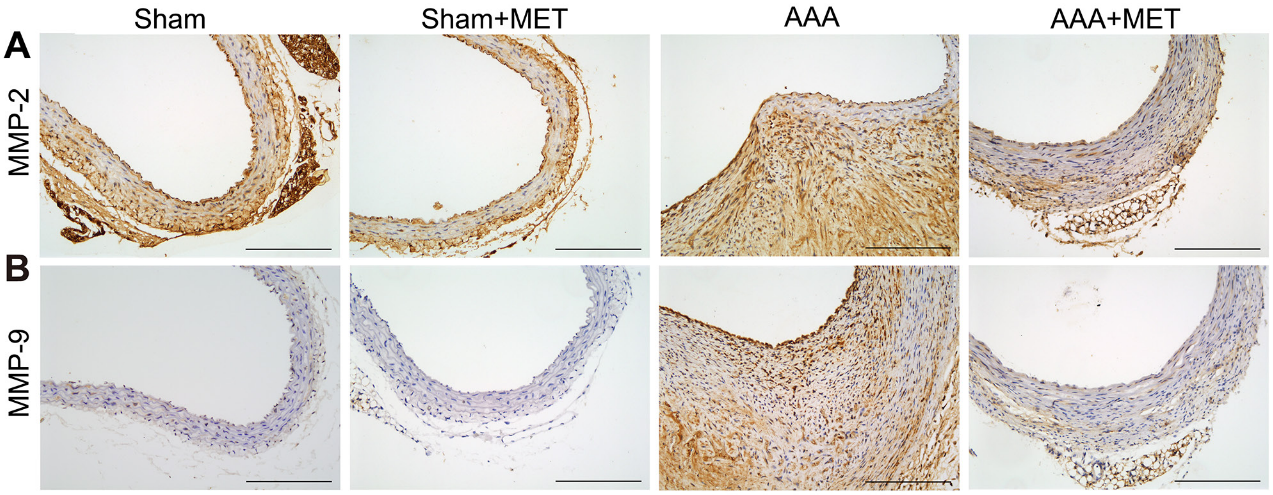

Supplementary Figure 4: Representative photomicrographs of MMP-2 **(A),** MMP-9 **(B)** staining of abdominal aortas of 4 groups of mice. Scale bar indicated 200 μm.

Supplementary Table 1: Primers used for qRT-PCR analysis

	Forward	Reverse
VEGFA	5’-GCACATAGGAGAGATGAGCTTCC-3’	5’-CTCCGCTCTGAACAAGGCT-3’
FLT-1	5’-TGCCTCTGAAGTTAGCCGTG-3’	5’-AGTTCTCCGCCGCCTTTTTA-3’
CD31	5’-ACTATGGCCTCAACCTGCTCATTC-3’	5’ -CTGGCATGATCTCAGCCACAA-3’
MCP-1	5’-GCATCCACGTGTTGGCTCA-3	5’-CTCCAGCCTACTCATTGGGATCA-3’
IL-1β	5’-TCCAGGATGAGGACATGAGCAC-3’	5’-GAACGTCACACACCAGCAGGTTA-3’
IL-6	5’-CCACTTCACAAGTCGGAGGCTTA-3’	5’-GCAAGTGCATCATCGTTGTTCATAC-3’
TNF-α	5’-AAGCCTGTAGCCCACGTCGTA-3’	5’-GGCACCACTAGTTGGTTGTCTTTG-3’
MMP-2	5’-GATAACCTGGATGCCGTCGTG-3’	5’-CTTCACGCTCTTGAGACTTTGGTTC-3’
MMP-9	5’-CATTCGCGTGGATAAGGAGT-3’	5’-ACCTGGTTCACCTCATGGTC-3’
GAPDH	5’-CATCCGTAAAGACCTCTATGCCAAC-3’	5’-ATGGAGCCACCGATCCACA-3’

Supplementary Table 2: Characteristics of AAA patients

Characteristics	Value (results are mean ± S.E.M)
Age (y)	63.6 ± 5.21
Sex (male/female)	13/2
Body mass index (kg/m^2)	21.3 ± 2.52
Diameter (cm)	6.03 ± 0.82
Smoking	9 (60%)
Diabetes	0
Hypertension	5 (33%)
Statin	3 (20%)
Metformin	0

Supplementary Table 3: Body mass, systolic blood pressure and heart rate of different groups of mice

**Body mass and haemodynamics**				
Results are means ± S.E.M				
**28 days after AAA induction**				
**Parameter**	**Sham**	**AAA**	**AAA+C.C**	**AAA+AICAR**
Body mass (g)	20.3±0.11	20.2±0.08	20.3±0.12	20.2±0.11
Systolic BP (mmHg)	102.7±8.4	115.7±8.8*	118.3±7.5*	117.5±8.3*
HR (beats/min)	557.6±36.8	552.3±26.8	561.7±32.6	547.8±35.2

*P<0.05, compared with Sham group.

Supplementary Table 4: Body mass, systolic blood pressure and heart rate of different groups of mice

**Body mass and haemodynamics**				
Results are means ± S.E.M				
**28 days after AAA induction**				
**Parameter**	**sham**	**Sham+met**	**AAA**	**AAA+met**
Body mass (g)	20.5±0.15	20.4±0.12	20.3±0.17	20.4±0.14
Systolic BP (mmHg)	105.7±7.4	107.5±7.9	117.8±6.4*	118.5±7.5*
HR (beats/min)	558.3±27.8	547.3±33.7	563.5±32.6	539.8±31.2

*P<0.05, compared with Sham group.
